# Regorafenib alone or in combination with high/low-dose radiotherapy plus toripalimab as third-line treatment in patients with metastatic colorectal cancer: protocol for a prospective, randomized, controlled phase II clinical trial (SLOT)

**DOI:** 10.3389/fonc.2023.1274487

**Published:** 2023-10-05

**Authors:** Shujuan Zhou, Chenchen Wang, Lijun Shen, Yan Wang, Hui Zhang, Ruiyan Wu, Yaqi Wang, Yajie Chen, Yan Xuan, Fan Xia, Zhen Zhang, Juefeng Wan

**Affiliations:** ^1^ Department of Radiation Oncology, Fudan University Shanghai Cancer Center, Shanghai, China; ^2^ Department of Oncology, Shanghai Medical College, Fudan University, Shanghai, China; ^3^ Shanghai Clinical Research Center for Radiation Oncology, Fudan University Shanghai Cancer Center, Shanghai, China; ^4^ Shanghai Key Laboratory of Radiation Oncology, Fudan University Shanghai Cancer Center, Shanghai, China; ^5^ Department of Gastrointestinal Medical Oncology, Fudan University Shanghai Cancer Center, Shanghai, China

**Keywords:** metastatic colorectal cancer, regorafenib, immunotherapy, low-dose radiotherapy, clinical protocol

## Abstract

Combination strategies to improve immunotherapy response in microsatellite stable metastatic colorectal cancer (MSS mCRC) remain an unmet need. Several single-arm clinical trials have shown promising synergistic effects between regorafenib and ICIs; however, some contradictory results have also been reported. Randomized controlled trials are needed to further validate the combination of regorafenib with ICIs. In addition, low-dose radiotherapy has been demonstrated to induce local immune responses by reprogramming the tumor microenvironment when combined with high-dose radiotherapy and ICIs. In this study, we designed a prospective, randomized, controlled phase II trial to investigate the efficacy and safety of regorafenib in combination with high/low-dose radiotherapy plus toripalimab in MSS mCRC compared to regorafenib alone. Patients with MSS metastatic adenocarcinoma of the colon or rectum will be enrolled and randomly assigned into two arms: a control arm and an experimental arm. Patients in the control arm will receive regorafenib monotherapy (120 mg once daily on days 1-21 of each 28 days cycle). Patients in the experimental arm will first receive one cycle of regorafenib (80 mg once daily on days 1-21 of each 28 days cycle) and toripalimab (240mg, q3w), followed by high-dose (4-8 fractions of 8-12Gy) and low-dose (1-10Gy at 0.5-2Gy/fraction) radiotherapy, and then continue regorafenib and toripalimab treatment. The primary endpoint is the objective response rate, and the secondary endpoints are disease control rate, duration of remission, median progress-free survival, median overall survival, and adverse events. Recruitment started in August 2023 and is ongoing.

**Clinical Trial Registration:**

https://clinicaltrials.gov/study/NCT05963490?cond=NCT05963490&rank=1, identifier NCT05963490.

## Introduction

Colorectal cancer (CRC) is the third most common cancer and the second most frequent cause of cancer deaths. Approximately 20% of patients with CRC have metastases at the time of diagnosis, and more than 50% of patients with CRC eventually develop metastases during their disease course (1). 95% of patients with metastatic CRC (mCRC) are microsatellite stable (MSS)/DNA mismatch repair proficient (pMMR) and unresponsive to immune checkpoint inhibitors (ICIs). More therapeutic options are needed to improve the outcomes of MSS mCRC patients.

Regorafenib is an oral multi-kinase inhibitor that targets signaling pathways involved in tumor angiogenesis (VEGFR1-3 and TIE2), oncogenesis (KIT, RET, RAF1, and BRAF), and the tumor microenvironment (PDGFR, FGFR, and CSF1R). It is currently approved as a salvage-line treatment for mCRC patients, but the objective response rate (ORR) is only 1%-4% (2, 3). Several mechanisms whereby regorafenib synergizes with ICIs have been evidenced by preclinical studies, including (1) reducing TAMs in tumors and modulating M1-like TAM polarization through inhibition of CSF1R (2); promoting trafficking and activity of effector T cells while decreasing recruitment of Tregs and MDSCs by targeting VEGFR (3); suppressing expression of PD-L1 and IDO1 by targeting the RET pathway (4). Multiple single-arm clinical trials have tested multi-kinase inhibitors such as regorafenib in combination with ICIs in mCRC and some of them have reported encouraging outcomes. The most impressive results were achieved in the REGONIVO study, with the ORR, median progress-free survival (mPFS), and median overall survival (mOS) of 33.3%, 7.9 months, and not reached, respectively (5). However, no objective responses were also observed when regorafenib was combined with pembrolizumab or avelumab (4). Therefore, the combination of regorafenib with ICIs warrants further validation in randomized, controlled trials.

Stereotactic ablative radiotherapy (SABR) is an effective local modality for the treatment of cancer metastases. Besides directly killing cancer cells, SABR simultaneously mobilizes innate and adaptive antitumor immune responses through (1) release of tumor antigens and damage-associated molecular molecules (DAMPs) (2); activation of the dsDNA-cGAS-STING pathway resulting in IFN production and DC maturation to prime tumor-specific T cells (3); secretion of cytokines and chemokines to promote T cell infiltration (6). A combination of SBRT and ICIs may synergistically unleash systemic T-cell responses and lead to abscopal effect. A phase II trial combining radiation of 8Gy×3Fx, ipilimumab, and nivolumab to treat patients with MSS mCRC reported a disease control rate (DCR) of 37% and an ORR of 15% outside of the irradiated field (7). This study provided proof of concept that SABR can increase the likelihood of responses to ICIs in MSS mCRC.

The clinical trials of low-dose radiotherapy (LDRT) started from the early 1930s in which patients with hematological or disseminated solid cancers were exposed to whole-and half-body LDRT totaling around 10Gy (8). The improved understanding of the interactions between radiation and the immune system has revived interest in LDRT’s potential to enhance immunotherapy. Recent studies showed that LDRT could reprogram the TME through (1) inflaming tumors with an influx of T cells including those newly primed by SABR, monocytes, DCs, and NK cells (2); reversing the immunosuppressive microenvironment with M1-like macrophage polarization, TGF-β decrease, and Treg reduction (9–11). That is to say, LDRT might augment local responses to immunotherapy and further boost abscopal effect rates when added to the combination of SABR and ICIs. The *post-hoc* analysis of patients who received LDRT either unintentionally as scatter or intentionally from three prospective immune-radiation trials reported an ORR of 58% in low-dose lesions compared with 18% in no-dose lesions (12). A phase II trial examined immunotherapy plus high-dose radiotherapy (HDRT) with or without LDRT for metastatic NSCLC and melanoma. The ORR in the HDRT+LDRT cohort was 26%, which doubled that in the HDRT-only cohort. The lesion-specific response was significantly improved in low-dose lesions (53%) compared with no-dose lesions in the both HDRT+LDRT (23%) and HDRT cohort (11%) (13). These studies provide foundations for combining SABR and LDRT as a potentially paradigm-changing approach in patients with larger, diffuse, or previously radiated metastases.

Based on the above rationale, we are conducting a randomized, controlled phase II trial to compare the efficacy and safety of regorafenib alone or in combination with SABR and LDRT plus toripalimab in MSS mCRC. Correlative studies will also be performed to explore potential predictive biomarkers and resistance mechanisms and inform better design of future clinical trials.

## Methods and analysis

### Study design

**Figure 1:**
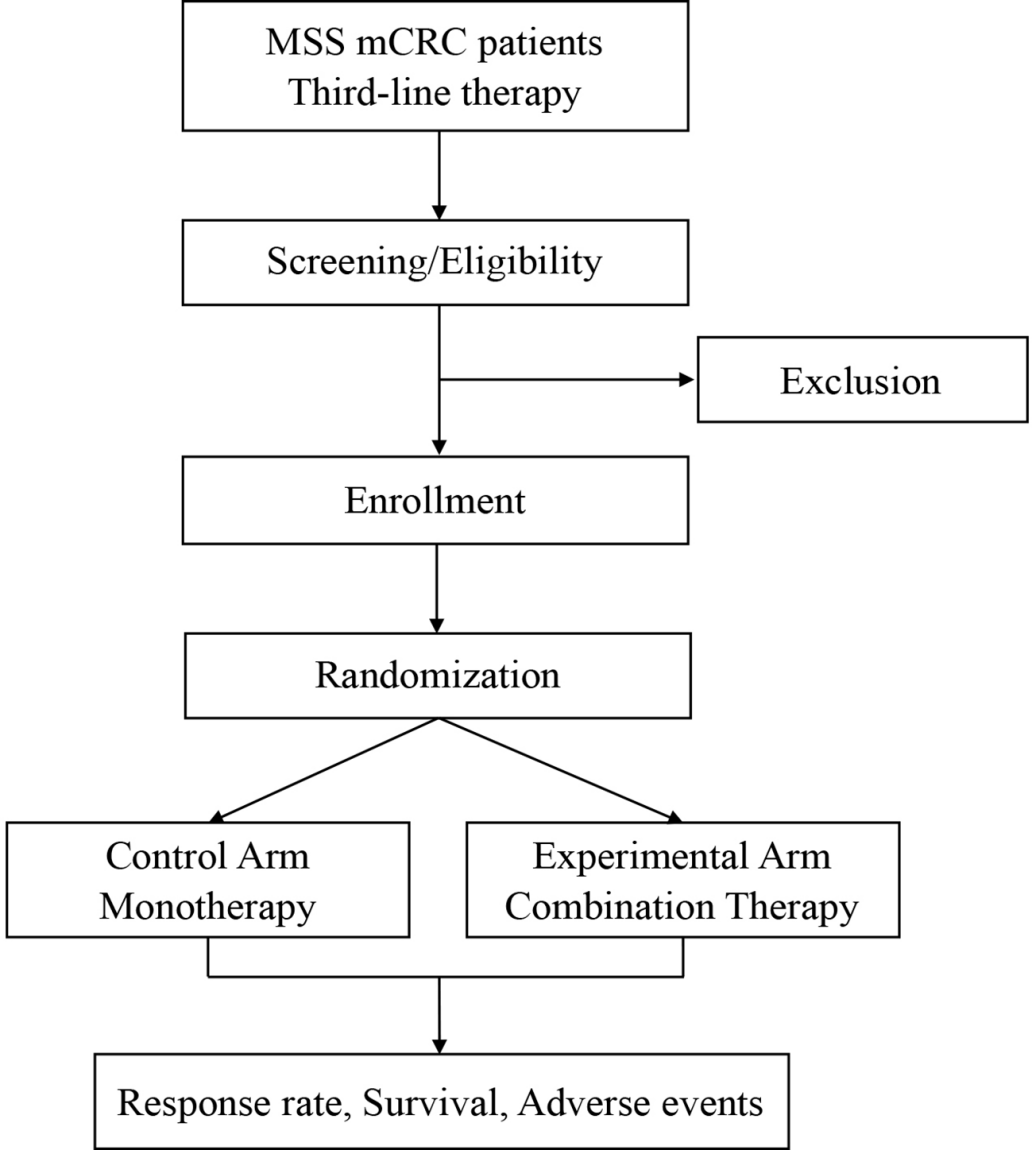
Study design of the SLOT trial.

SLOT is a prospective, randomized, controlled, investigator-initiated phase II trial carried out at Fudan University Shanghai Cancer Center (FUSCC) in China. This is to our knowledge the first prospective trial investigating a novel radiotherapy regimen with SABR and LDRT in mCRC in addition to ICI and regorafenib. Patients with metastatic MSS CRC, who have failed or are intolerant of the standard first-and second-line therapies, will be enrolled and randomly assigned into two arms: a control arm and an experimental arm. Patients in the control arm will receive regorafenib monotherapy. Patients in the experimental arm will first receive one cycle of regorafenib and toripalimab, followed by SABR and LDRT radiotherapy, and then continue regorafenib and toripalimab treatment. The ORR, DCR, duration of remission (DoR), mPFS, mOS, and adverse effects will be analyzed. The study algorithm is presented in **Figure 1**.

### Key eligibility criteria

Eligible patients should present histologically confirmed, MSS, metastatic (at least two evaluable lesions) CRC and have received two prior lines of chemotherapy. In addition, patients are required to have good performance status, normal organ function, no active autoimmune disease or infections, and no history of ICI or regorafenib treatment. Previous radiotherapy performed at least 4 weeks before enrollment is allowed. Inclusion and exclusion criteria are listed in detail in **Table 1**.

**Table 1:** Inclusion and exclusion criteria.

Inclusion criteria	Exclusion criteria
1. Age ≥18 years old 2. An Eastern Cooperative Oncology Group (ECOG) performance status **≤**1 3. Life expectancy of at least 3 months 4. Histopathological confirmed MSS/pMMR adenocarcinoma of the colon or rectum 5. At least two evaluable metastatic lesions for SABR and LDRT according to RECIST 1.1 6. Progressed on or after the standard first-and second-line therapies or stopped standard therapy because of unacceptable toxic effects 7. Previous radiotherapy completed at least 4 weeks before randomization 8. Adequate bone-marrow, hepatic, and renal function: neutrophils ≥ 1.5 × 10^9/L, Hb ≥ 90 g/L, PLT ≥ 100 × 10^9/L, ALT**/**AST **≤** 2.5 ULN, Cr **≤** 1 ULN 9. Sign the informed consent and have good compliance	1. History of previous treatment with regorafenib and ICIs such as anti-PD-1 or anti-PD-L1 mAbs 2. Current severe cardiovascular diseases such as unstable angina, congestive heart failure, or serious cardiac arrhythmia requiring medication 3. Acute cardiac infarction or cerebral ischemic stroke occurred within 6 months before recruitment 4. Active autoimmune diseases and immunodeficiencies, known history of organ transplantation, or systematic use of immunosuppressive agents 5. Active Hepatitis B virus (HBV) or hepatitis C virus (HCV) infection: HBsAg positive or HBV DNA positive, anti-HCV antibody testing positive and confirmatory HCV RNA positive 6. Positive human immunodeficiency virus (HIV) infection, active syphilis infection, or active pulmonary tuberculosis infection 7. Severe infections requiring systemic antibiotics, antifungal or antiviral therapy 8. Uncontrollable pleural effusion, pericardial effusion, or ascites 9. Other malignancies within 5 years before recruitment, except for non-melanoma skin cancer, superficial bladder cancer, cervical carcinoma *in situ*, or breast cancer *in situ* that had been effectively treated. 10. Known history of severe neurological or mental illness such as schizophrenia, dementia, or epilepsy 11. Known history of allergy to any component in this study. 12. Pregnancy or breast-feeding women

### Randomization process

Eligible patients will be randomly assigned into the control arm with regorafenib monotherapy and the experimental arm with combination therapies in a 1:1 proportion. Randomization is performed via a secure software based on a stratified blocked randomization design. The block size is also randomized and the stratification factor is the presence of liver metastases (yes vs no). The result of randomization is immediately available in the software and will be forwarded to the investigator.

### Interventions

**Figure 2:**
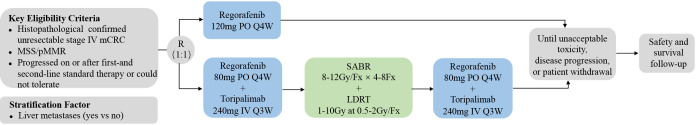
Flowchart of the SLOT trial.

Eligible patients will receive treatment as follows (1): control arm: regorafenib 120 mg orally once daily on days 1-21 of each 28 days cycle (2). experimental arm: patients will first receive one cycle of regorafenib and toripalimab followed by SABR/LDRT radiotherapy. Regorafenib and toripalimab will be continued after the completion of radiotherapy. In this arm, regorafenib is administered 80 mg once daily on days 1-21 of each 28 days cycle with intravenous toripalimab 240 mg every 3 weeks. Radiotherapy regimes include 4-8 fractions of 8-12Gy via SABR and up to 1-10Gy at 0.5-2Gy/fraction via LDRT (**Figure 2**). Patients will follow the treatment program until disease progression, unacceptable toxic effects, or withdrawal of consent.

Regorafenib: dose modifications are permitted to manage clinically significant treatment-related adverse events (AEs) of regorafenib. If AEs of grade 3 occur, regorafenib will be suspended and the AEs will be managed until the AEs have been resolved to grade 1 or baseline levels. The regorafenib dose may be reduced by 40 mg for the next treatment at the discretion of the investigator. Regorafenib will be discontinued permanently if the toxic effect did not recover after a 4-week interruption, after two consecutive dose reductions in the control arm and one dose reduction in the experimental arm (minimum permissible dose 40 mg per day), or if there are AEs of grade 4.

Radiotherapy: Radiotherapy will be initiated 3-7 days after the first administration of toripalimab, depending on actual time intervals from simulation localization to irradiation. Patients will first receive SABR and then LDRT after the completion of SABR. LDRT will be delivered using intensity-modulated radiotherapy (IMRT) or volumetric-modulated arc therapy (VMAT) and will target large tumors, previously radiated tumors, and those located in areas more sensitive to radiation’s adverse effects. The choice of radiotherapy regimen is left to the discretion of the physician. For metastatic lesions in the liver, lungs, bones, and brain, the radiation field only includes gross tumor volumes (GTV) plus a 5-10 mm margin. Regional prophylactic irradiation of the lymph node drainage area may also be performed for metastatic lymph nodes. Four-dimensional CT (4DCT), passive breath gating (PBG), and abdominal spatula will be adopted to eliminate the influence of respiratory movement.

Immunotherapy: the ICI used in this study will be toripalimab which is administrated intravenously at 240 mg on day 1 of every 3-week cycle. Toripalimab is provided free of charge for the first three cycles by Shanghai Junshi Biomedical Technology Co., Ltd., which has also purchased liability insurance for clinical trial subjects.

### Endpoints

**Figure 3:**
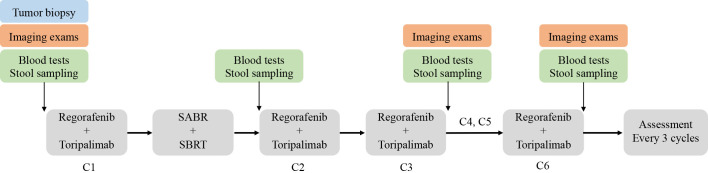
Timeline for assessment of the SLOT trial.

The primary endpoint of this study is ORR according to RECIST 1.1, and the secondary endpoints are DCR, DoR, mPFS, mOS, and adverse events. Exploratory objectives include potential predictive biomarkers and resistance mechanisms. Therefore, we will conduct genomic (DNA and RNA) sequencing and multicolor immunohistochemical staining using baseline tumor biopsies, FACS analyses of peripheral leukocytes and plasma cytokines using blood samples, and 16S rRNA sequencing of the gut microbiome using stool samples. Time points for sample collection are described in **Figure 3**.

### Assessment and follow up

Regular radiological examinations consisting of pelvic MRI/CT, abdominal MRI/CT, chest CT, head MRI, or PET-CT (depending on the locations of metastases) will be conducted before treatment initiation and after every three cycles of toripalimab. Imaging efficacy is evaluated according to the Response Evaluation Criteria in solid tumors (RECIST v.1.1). The primary endpoint ORR and secondary DCR will be assessed based on LDRT-treated lesions in patients without non-irradiated lesions. For patients with non-irradiated lesions, ORR and DCR will be evaluated based on both LDRT-treated lesions and measurable non-irradiated lesions. Patients will also receive blood tests at baseline, after the completion of radiotherapy but before the second toripalimab, and after every three cycles of toripalimab. These tests include serum tumor markers (CEA, AFP, CA724, CA242, CA199, etc.), immune-related indicators (myocardial enzymes, thyroid hormones, and adrenal hormones), serum cytokines (IL-2, IL-6, IL-10, IFN-γ, etc.), and PBMC immunophenotyping of both lymphoid and myeloid cells (**Figure 3**). Adverse events will be evaluated throughout the treatment period and recorded according to the National Cancer Institute Common Terminology Criteria for Adverse Events (CTCAE) v5.0. Quality of life during the treatment and follow-up period will be evaluated using the EORTC QLQ-C30 and EORTC QLQ-CR29 scales.

Patients who discontinue the protocol treatment will be scheduled for survival follow-up by telephone or clinic visits every two months for one year, every three months in the second year, every six months in the third to fifth year, and once a year thereafter. Subsequent therapy after progression, the cause of death, and the date of death or last follow-up visit will be recorded in detail.

### Sample size

This is a prospective, randomized, controlled phase II trial and the primary endpoint is ORR. The CONCUR trial showed that regorafenib monotherapy yielded an ORR of 4%. A study of regorafenib plus toripalimab (NCT03946917) reported an ORR of 15.2% and a study combining high/low-dose radiotherapy and ICIs (NCT02710253) showed an ORR of 26% (13, 14). Based on these results, the ORR in the control arm is set as 4% (P1), and we assume that the ORR in the experimental arm can be increased to 25% (P2). The sample size is calculated using Z-test in PASS 2021. Patients will be randomly assigned into two arms at a ratio of 1:1. With a one-sided significance level (α) of 0.05 and statistical power (1-β) of 80%, 64 patients (32 patients per arm) need to be enrolled. Taking into account a maximum dropout rate of 10%, the final total sample size in this study will be 70 cases (35 cases per arm).

### Statistical analysis

In this study, the SPSS 2021 software will be used for statistical analysis. Objective response and disease control rates between treatment groups will be compared using the Cochran-Mantel-Haenszel test adjusted for stratification factors. Fisher’s exact test will be used to compare the patient characteristics between the two arms. Duration of Response, overall survival, and progression-free survival for each arm will be estimated using the Kaplan-Meier method. We will compare survival using a stratified log-rank test, and calculate HRs (with 95% CIs) using the Cox model, adjusting for baseline stratification factors. All statistical tests are two-sided and the level of significance is P < 0.05.

## Discussion

The CORRECT and CONCUR study showed an ORR of 0%-4% and an mOS of 6.4-8.8 months with regorafenib monotherapy in MSS mCRC, which left much room for improvement. The efficacy data reported in multiple single-arm phase II clinical trials appear to favor regorafenib in combination with ICIs over either single agent, with ORR and mOS ranging between 7.1%-33.3% and 9.6-15.5 months (or not reached), respectively (4). However, two studies combining regorafenib with pembrolizumab or avelumab demonstrated an 0% of ORR (15, 16). Such inconsistency may have resulted from heterogeneity across studies in terms of geographic region, patient characteristics (especially treatment line and the proportion of patients with liver metastases), sample size, etc. What’s more, there is a lack of randomized, controlled trials to provide the most robust evidence about the relative efficacy of regorafenib in combination with ICIs versus regorafenib alone. Therefore, we conduct the current study to further validate the efficacy and safety of combining regorafenib with toripalimab.

Besides synergizing with ICIs, regorafenib may also enhance the radiosensitivity of colorectal tumors by blocking radiation-induced activation of receptor tyrosine kinases, inhibiting VEGF-mediated angiogenesis, and suppressing DNA damage repair (17, 18). However, only a few studies have demonstrated these effects of regorafenib using tumor cell lines and mouse models, and no clinical trials investigating regorafenib in combination with radiotherapy have been reported. This is probably because the radiosensitization effect of regorafenib is not obvious enough; thus, combinations with other anticancer agents like ICIs are needed.

Different radiation doses vary in immunomodulatory effects. 8-12Gy high-dose radiation modulates systemic immune responses by facilitating tumor antigen release, presentation, and recognition to prime T cells which circulate throughout the body. Low-dose radiation mainly modulates the local TME by recruiting effector cells and reducing immunosuppressive stroma components (19). Recently, a pilot study (RACIN, NCT03728179) testing the efficacy of LDRT in combination with ICIs reported an ORR of 12.5% in eight patients with ‘cold’ solid tumors, and two additional patients achieved dramatic responses by PET (11). Despite this, we recommend the combined high-dose and low-dose radiotherapy plus ICIs as a better treatment regimen, based on the results of phase II studies (NCT0271025 and NCT02710253) and retrospective analysis (12, 13, 20, 21). The assumption is that high-dose radiation primes tumor-reactive T cells, while low-dose radiation facilitates the infiltration of these T cells into irradiated sites. Of note, we emphasize the importance of high-dose radiation to liver metastases which delete T cells and diminish immunotherapy efficacy (22). We also suggest low-dose radiation to all metastatic deposits, as tumor progressions were observed only outside the irradiated field in the RACIN study (11).

Overall, this prospective, randomized, controlled phase II trial investigates whether the addition of high/low-dose radiotherapy and immunotherapy can achieve better responses and prolonged survival with good tolerance compared to regorafenib monotherapy, aiming to provide an effective treatment strategy for patients with MSS mCRC.

## Data availability statement

The original contributions presented in the study are included in the article/supplementary material. Further inquiries can be directed to the corresponding authors.

## Ethics statement

The studies involving humans were approved by the Ethics Committee of Fudan University Shanghai Cancer Center. The studies were conducted in accordance with the local legislation and institutional requirements. The participants provided their written informed consent to participate in this study.

## Author contributions

SZ: Formal Analysis, Methodology, Software, Writing – original draft, Writing – review & editing. CW: Funding acquisition, Supervision, Writing – review & editing. LS: Investigation, Resources, Supervision, Writing – original draft. YanW: Project administration, Resources, Supervision, Writing – original draft. HZ: Data curation, Investigation, Project administration, Supervision, Writing – original draft. RW: Data curation, Investigation, Project administration, Writing – original draft. YaqW: Data curation, Software, Writing – original draft. YC: Investigation, Methodology, Project administration, Writing – original draft. YX: Data curation, Formal Analysis, Investigation, Writing – original draft. FX: Project administration, Supervision, Validation, Writing – review & editing. ZZ: Conceptualization, Supervision, Validation, Visualization, Writing – review & editing. JW: Conceptualization, Supervision, Validation, Visualization, Writing – review & editing.

## References

[1] Ciardiello F Ciardiello D Martini G Napolitano S Tabernero J Cervantes A . Clinical management of metastatic colorectal cancer in the era of precision medicine. CA Cancer J Clin (2022) 72(4):372–401. doi: 10.3322/caac.21728 35472088

[2] Li J Qin SK Xu RH Yau TCC Ma B Pan HM . Regorafenib plus best supportive care versus placebo plus best supportive care in Asian patients with previously treated metastatic colorectal cancer (CONCUR): a randomised, double-blind, placebo-controlled, phase 3 trial. Lancet Oncol (2015) 16(6):619–29. doi: 10.1016/S1470-2045(15)70156-7 25981818

[3] Grothey A Van Cutsem E Sobrero A Siena S Falcone A Ychou M . Regorafenib monotherapy for previously treated metastatic colorectal cancer (CORRECT): an international, multicentre, randomised, placebo-controlled, phase 3 trial. Lancet (2013) 381(9863):303–12. doi: 10.1016/S0140-6736(12)61900-X 23177514

[4] Akin Telli T Bregni G Vanhooren M Saude Conde R Hendlisz A Sclafani F . Regorafenib in combination with immune checkpoint inhibitors for mismatch repair proficient (pMMR)/microsatellite stable (MSS) colorectal cancer. Cancer Treat Rev (2022) 110:102460. doi: 10.1016/j.ctrv.2022.102460 36058142

[5] Fukuoka S Hara H Takahashi N Kojima T Kawazoe A Asayama M . Regorafenib plus nivolumab in patients with advanced gastric or colorectal cancer: an open-label, dose-escalation, and dose-expansion phase ib trial (REGONIVO, EPOC1603). J Clin Oncol (2020) 38(18):2053–61. doi: 10.1200/Jco.19.03296 32343640

[6] McLaughlin M Patin EC Pedersen M Wilkins A Dillon MT Melcher AA . Inflammatory microenvironment remodelling by tumour cells after radiotherapy. Nat Rev Cancer (2020) 20(4):203–17. doi: 10.1038/s41568-020-0246-1 32161398

[7] Parikh AR Szabolcs A Allen JN Clark JW Wo JY Raabe M . Radiation therapy enhances immunotherapy response in microsatellite stable colorectal and pancreatic adenocarcinoma in a phase II trial. Nat Cancer (2021) 2(11):1124–35. doi: 10.1038/s43018-021-00269-7 PMC8809884 35122060

[8] Janiak MK Pociegiel M Welsh JS . Time to rejuvenate ultra-low dose whole-body radiotherapy of cancer. Crit Rev Oncol Hematol (2021) 160:103286. doi: 10.1016/j.critrevonc.2021.103286 33667656

[9] Herrera FG Romero P Coukos G . Lighting up the tumor fire with low-dose irradiation. Trends Immunol (2022) 43(3):173–9. doi: 10.1016/j.it.2022.01.006 35105519

[10] Klug F Prakash H Huber PE Seibel T Bender N Halama N . Low-dose irradiation programs macrophage differentiation to an iNOS(+)/M1 phenotype that orchestrates effective T cell immunotherapy. Cancer Cell (2013) 24(5):589–602. doi: 10.1016/j.ccr.2013.09.014 24209604

[11] Herrera FG Ronet C Ochoa de Olza M Barras D Crespo I Andreatta M . Low-dose radiotherapy reverses tumor immune desertification and resistance to immunotherapy. Cancer Discov (2022) 12(1):108–33. doi: 10.1158/2159-8290.CD-21-0003 PMC9401506 34479871

[12] Menon H Chen D Ramapriyan R Verma V Barsoumian HB Cushman TR . Influence of low-dose radiation on abscopal responses in patients receiving high-dose radiation and immunotherapy. J Immunother Cancer (2019) 7(1):237. doi: 10.1186/s40425-019-0718-6 31484556 PMC6727581

[13] Patel RR He KW Barsoumian HB Chang JY Tang C Verma V . High-dose irradiation in combination with non-ablative low-dose radiation to treat metastatic disease after progression on immunotherapy: Results of a phase II trial. Radiotherapy Oncol (2021) 162:60–7. doi: 10.1016/j.radonc.2021.06.037 PMC11905861 34237343

[14] Wang F He MM Yao YC Zhao X Wang ZQ Jin Y . Regorafenib plus toripalimab in patients with metastatic colorectal cancer: a phase Ib/II clinical trial and gut microbiome analysis. Cell Rep Med (2021) 2(9):100383. doi: 10.1016/j.xcrm.2021.100383 34622226 PMC8484502

[15] Cousin S Cantarel C Guegan JP Gomez-Roca C Metges JP Adenis A . Regorafenib-avelumab combination in patients with microsatellite stable colorectal cancer (REGOMUNE): A single-arm, open-label, phase II trial. Clin Cancer Res (2021) 27(8):2139–47. doi: 10.1158/1078-0432.CCR-20-3416 33495314

[16] Barzi. A Azad. NS Yang. Y Tsao-Wei. D Rehman. R Fakih. M . Phase I/II study of regorafenib (rego) and pembrolizumab (pembro) in refractory microsatellite stable colorectal cancer (MSSCRC). J Clin Oncol (2022) 40(4_suppl):15. doi: 10.1200/JCO.2022.40.4_suppl.015

[17] Liu YC Chiang IT Chung JG Hsieh JH Chiang CH Weng MC . Therapeutic efficacy and inhibitory mechanism of regorafenib combined with radiation in colorectal cancer. In Vivo (2020) 34(6):3217–24. doi: 10.21873/invivo.12157 PMC7811665 33144426

[18] Mehta M Griffith J Panneerselvam J Babu A Mani J Herman T . Regorafenib sensitizes human breast cancer cells to radiation by inhibiting multiple kinases and inducing DNA damage. Int J Radiat Biol (2021) 97(8):1109–20. doi: 10.1080/09553002.2020.1730012 PMC7882427 32052681

[19] Ji X Jiang W Wang J Zhou B Ding W Liu S . Application of individualized multimodal radiotherapy combined with immunotherapy in metastatic tumors. Front Immunol (2022) 13:1106644. doi: 10.3389/fimmu.2022.1106644 36713375 PMC9877461

[20] Barsoumian HB Ramapriyan R Younes AI Caetano MS Menon H Comeaux NI . Low-dose radiation treatment enhances systemic antitumor immune responses by overcoming the inhibitory stroma. J Immunother Cancer (2020) 8(2):e000537. doi: 10.1136/jitc-2020-000537 33106386 PMC7592253

[21] Yin LM Xue JX Li R Zhou L Deng L Chen L . Effect of low-dose radiation therapy on abscopal responses to hypofractionated radiation therapy and anti-PD1 in mice and patients with non-small cell lung cancer. Int J Radiat Oncol (2020) 108(1):212–24. doi: 10.1016/j.ijrobp.2020.05.002 32417411

[22] Yu J Green MD Li S Sun Y Journey SN Choi JE . Liver metastasis restrains immunotherapy efficacy via macrophage-mediated T cell elimination. Nat Med (2021) 27(1):152–64. doi: 10.1038/s41591-020-1131-x PMC8095049 33398162

